# Hydrophilic and Conductive Carbon Nanotube Fibers for High-Performance Lithium-Ion Batteries

**DOI:** 10.3390/ma14247822

**Published:** 2021-12-17

**Authors:** Nayoung Ku, Jaeyeong Cheon, Kyunbae Lee, Yeonsu Jung, Seog-Young Yoon, Taehoon Kim

**Affiliations:** 1 Composites Research Division, Korea Institute of Materials Science, Changwon 51508, Korea; nayoung@kims.re.kr (N.K.); jycheon@kims.re.kr (J.C.); kblee@kims.re.kr (K.L.); ysjung@kims.re.kr (Y.J.); 2 School of Materials Science and Engineering, Pusan National University, Busan 46241, Korea

**Keywords:** CNT fiber, yarn, lithium-ion battery, anode, tin oxide

## Abstract

Carbon nanotube fiber (CNTF) is a highly conductive and porous platform to grow active materials of lithium-ion batteries (LIB). Here, we prepared SnO_2_@CNTF based on sulfonic acid-functionalized CNTF to be used in LIB anodes without binder, conductive agent, and current collector. The SnO_2_ nanoparticles were grown on the CNTF in an aqueous system without a hydrothermal method. The functionalized CNTF exhibited higher conductivity and effective water infiltration compared to the raw CNTF. Due to the enhanced water infiltration, the functionalized CNTF became SnO_2_@CNTF with an ideal core–shell structure coated with a thin SnO_2_ layer. The specific capacity and rate capability of SnO_2_@-functionalized CNTF were superior to those of SnO_2_@raw CNTF. Since the SnO_2_@CNTF-based anode was free of a binder, conductive agent, and current collector, the specific capacity of the anode studied in this work was higher than that of conventional anodes.

## 1. Introduction

Fascinating physical properties of carbon nanotubes (CNTs), including their high strength, high electrical conductivity, and high specific surface area, have driven researchers to use CNTs in electronic applications for more than 20 years [1,2]. Remarkable commercial growth is just approaching as CNTs are being used in electrodes of lithium-ion battery (LIB) as a conductive additive [3]. Due to the high electrical conductivity and one-dimensional morphology of the CNTs [4], the incorporation of a small amount can effectively form an electron pathway in the electrode active materials, resulting in high energy density. Indeed, the conductive additives of LIB electrodes have engendered a new era of CNT. The next step will be replacing current collectors of the LIB with CNTs. This can boost the energy density of the LIB by reducing the weight of current collectors. However, it is hard to achieve conductivity as high as that of metal current collectors based on mixing the CNTs with dispersion agents, binding polymers (polyvinylidene fluoride, carboxymethyl cellulose, etc.), and active materials (graphite, silicon, etc.).

CNT fiber (CNTF) is a macroscopic CNT assembly that is one of the promising candidates to replace the current collector [5,6] of LIB because of its high electrical conductivity and mechanical strength [7]. CNTF is distinguished from conventional CNT assemblies such as CNT films prepared from dispersed suspensions and CNT buckypapers prepared by a filtering method because no dispersion agent or binders are incorporated in the CNTF. Among the three proposed synthesis methods (i.e., forest-spinning method, wet-spinning method, and direct-spinning methods), CNTFs prepared by the direct-spinning method have both high porosity and conductivity simultaneously [7,8]. Those features make the CNTF an appropriate conductive platform for the incorporation of active materials in the pores of CNTF to be used in an energy storage system. If nanoparticles of silicon, graphite, lithium titanium oxide, or tin oxide are incorporated in the nanopores of the CNTF, then current collectors, binding polymers, and conductive agents are no longer required. Indeed, current collector-free and binder-free supercapacitors and LIB systems have been reported recently based on direct-spun CNTF [9,10,11].

The hydrophobic nature of the CNT surface has been an issue that prevented dispersion of CNTs in an aqueous system. Since the aqueous system is environmentally benign and economically favorable for wastewater treatment compared with other organic solvent systems, many researchers have reported surface treatments of CNT to disperse the CNT in water. Functionalization such as the acid treatment of CNT was effective at dispersing CNT in the aqueous solvent [12]. Likewise, the intrinsically hydrophobic surface of CNTF prohibits penetration of water into the nanopores of CNTF, implying that the nanopores of CNTF cannot provide reaction sites for nanoparticle growth, electrochemical reactions, and catalytic reactions in an aqueous system. Therefore, enhancing the hydrophilicity of the CNTF by surface functionalization is necessary for preparing active materials-incorporated CNTF in an aqueous system. Recently, we reported a surface modification method for CNTF to improve the hydrophilicity of the fibers [13]. In particular, sulfanilic acid groups were more effective than the carboxyl group to enhance the hydrophilicity of CNTF.

In this work, we propose a current collector-free and conductive agent-free LIB anode based on hydrophilic CNTF and SnO_2_ (Tin oxide) nanoparticles fabricated in an aqueous system. SnO_2_ nanoparticles can afford high theoretical capacity (1494 mAh/g) [14,15] and be synthesized on nanocarbon in an aqueous system [16,17,18], offering an environmentally friendly preparation method for LIB anode materials. We first introduced the sulfanilic acid on CNTF for preparing functionalized CNTF with various degrees of functionalization to determine the most appropriate CNTF for growing SnO_2_ nanoparticles. Subsequently, anode materials were prepared by synthesizing the SnO_2_ nanoparticles on the functionalized CNTF, and the anode performance was examined. The SnO_2_@hydrophilic CNTF remarkably enhanced anode capacity compared with SnO_2_@raw CNTF because of the uniform growth of SnO_2_ nanoparticles and enhanced conductivity. This study provides new physical insight not only into preparing high-performance LIB anode materials, but into synthesizing hydrophilic and highly conductive CNTF that can be used for any type of electrode in an aqueous system.

## 2. Materials and Methods

### 2.1. Preparation of SnO_2_ Nanoparticles@CNTF

CNTF was synthesized under conditions in which only the amount of thiophene increased by the direct-spinning method described in the previous articles [19,20,21,22]. In short, carrier gases (i.e., argon and hydrogen), precursors (i.e., ferrocene and thiophene), and methane gas were injected into a vertical furnace at 1200 °C. CNT aerogel was shrunk into fiber in a water bath below the furnace and subsequently wound on a winder at 5 m/min. The CNTF was soaked in acetone and dried, which is denoted as raw CNTF.

We introduced sulfonic acid groups on the raw CNTF by a diazotization method, as we reported before [13,20]. The diazonium ions were prepared by a reaction of 0.025–0.3 M of sulfanilic acid (Sigma Aldrich, Burlington, MA, USA) and an equivalent amount of sodium nitrite (NaNO_2_, Sigma Aldrich) in 1 M HCl. Raw CNTF was soaked in the solutions for 2 h at 90 °C and washed in acetone. We named the functionalized CNTF as nS-CNTF, where n means the concentration of sulfanilic acid.

SnO_2_ nanoparticles were grown on the raw CNTF and functionalized CNTF. First, 12 g of tin chloride (SnCl_2_, anhydrous, Alfa Aesar, Ward Hill, MA, USA) were dissolved in the solution consisting of 150 mL deionized water and 1.5 mL hydrochloric acid (HCl, 37%, Sigma-Aldrich). The CNTF was soaked in the tin chloride solution for 8 h at 90 °C. After growing the SnO_2_ nanoparticles, the CNTF was washed in 1 M HCl and deionized water and heat-treated at 300 °C under Ar atmosphere. We named the SnO_2_@CNTF as nSC, where n means the concentration of sulfanilic acid during surface treatment. The 0SC refers to the SnO_2_@raw CNTF.

### 2.2. Characterization

The amount of sulfur in CNTF was characterized using X-ray photoelectron spectroscopy (XPS; K-Alpha+, ThermoFisher Scientific, Waltham, MA, USA). Electrochemical characterization was conducted using a VSP-300 potentiostat (BioLogic, Seyssinet-Pariset, France). To investigate electrochemical double-layer capacitance (EDLC) of the CNTF, electrochemical measurements were performed in a three-electrode configuration with CNTF as a working electrode in 0.15 M NaClO_4_ electrolyte. Graphite rod and Ag/AgCl (Sat. KCl, ALS RE-1CP) were used as counter and reference electrodes, respectively. EDLC of the CNTF was determined by cyclic voltammetry (CV) at the open circuit potential (OCP). After ~200 s of OCP stabilization, CV scans spanning ± 50 mV of the OCP were recorded at scan rates of 5, 10, 20, 30, 40, and 50 mV/s. The capacitive current was calculated by using the difference between the cathodic and anodic currents (*i_a_ − i_c_*) at the OCP. These capacitive currents were then plotted vs. scan rate, and the slope of this plot was divided by 2 to obtain the capacitance of CNTF at the OCP.

(1) $$\mathrm{Electrode}\;\mathrm{capacitance}\;=\;\frac{1}{2}\frac{\partial \left(i_{a}-i_{c}\right)}{\partial \left(scan\;rate\right)}$$

Morphologies of CNTF and SnO_2_@CNTF were analyzed by field emission scanning electron microscopy (FE-SEM, JSM-7001F, JEOL, Tokyo, Japan). The linear density of the CNTF was measured by weighing 10 m of CNTF using an excellence level balance (XPE205). The ratio of SnO_2_ of SnO_2_@CNTF was obtained from a comparison of linear density before and after growing SnO_2_ nanoparticles. The density of the CNTF was obtained from the calculation of a cross-sectional area and linear density of CNTF, which is 0.8 g/cm^3^ in this work. The specific tensile strength of the CNTF was characterized using a universal testing machine (3344, Instron, Norwood, MA, USA) with the ASTM C1557 method, as described in our previous article [23]. The gauge length of the sample was 1 cm and the crosshead speed was 0.3 mm/min.

### 2.3. Cell Testing

SnO_2_@CNTF was transferred into an Argon-filled glove box (O_2_, H_2_O < 1 ppm) for cell assembly. The length of the SnO2@CNTF was 14 cm with an aspect ratio of 2800. The 2032-type coin cells with SnO_2_@CNTF, 1 M LiPF_6_ in ethylene carbonate/diethylene carbonate (EC:DEC, 1:1 by volume), commercial celgard 2400 separator, and Li metal were assembled to assess the energy-storage capability of SnO_2_@CNTF. Prepared SnO_2_@CNTF was used directly as anode without any additive or further treatments.

Charge/discharge tests were performed on an electrochemical station (Automatic Battery Cycler, WBCS3000L, WonaTech, Seoul, Korea,) over 0.01–2.5 V (vs. Li/Li^+^). CV was performed by VSP-300 electrochemical station at 0.1 mV/s scan rate over 0.01–3.0 V (vs. Li/Li^+^); electrochemical impedance spectroscopy (EIS) was conducted using Electrochemical Workstation ZIVE SP1 (WonaTech) over 1 MHz to 10 mHz with 5-mV amplitude.

## 3. Results

**Figure 1:**
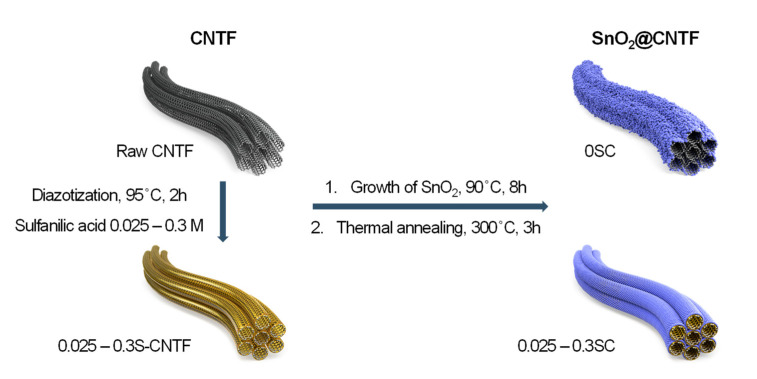
Schematic representation of preparation processes and name of the samples in this work.

Figure 1 illustrates a preparation scheme of SnO_2_@CNTF studied in this work. We prepared raw CNTF and surface-modified CNTF as conductive platform materials to grow SnO_2_ nanoparticles. The surface-modified CNTF was prepared by the diazotization method reported in previous works with further optimization [13,20]. We manipulated the degree of functionalization by changing the concentration of the sulfanilic acid to find optimum CNTF. SnO_2_ nanoparticles were grown on the surface of CNTF with neither organic solvents nor a hydrothermal system. This facile and environmentally benign synthesis method enables mass production at a low cost. Mild thermal annealing at 300 °C followed to increase the crystallinity of the SnO_2_ nanoparticles. The prepared materials were evaluated in a coin-cell system.

**Figure 2:**
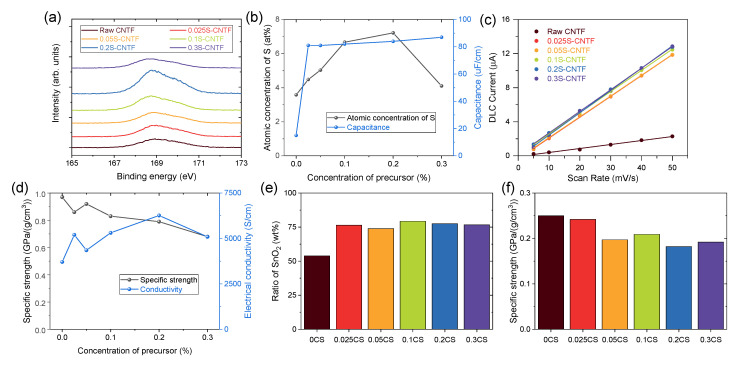
(**a**) S2p XPS spectra of CNTF and (**b**) quantitative analysis of sulfur and capacitance of the CNTF. (**c**) Capacitive currents of CNTF versus scan rate. (**d**) Specific strength and conductivity of CNTF. (**e**) Ratio of SnO_2_ and (**f**) specific strength of SnO_2_@CNTF.

S2p XPS spectra quantitatively showed the degree of functionalization of surface-modified CNTF (Figure 2a). The XPS spectra indicated that the raw CNTF also had sulfur atoms, because thiophene was used to grow CNTF in the direct-spinning process. In particular, we used a larger amount of sulfur precursor in this study to prepare the CNTF with high linear density [8,24], which resulted in the noticeable S2p peak of raw CNTF. The intensity of the S2p spectra increased as the concentration of sulfanilic acid used in the functionalization process increased except for the condition of 0.3 M sulfanilic acid. When the concentration of sulfanilic acid was too high, the sulfanilic acid remained undissolved until the reaction finished due to the low solubility of sulfanilic acid. The undissolved sulfanilic acid was deposited on the CNTF and prevented functionalization. Quantitative analysis of the atomic ratio of CNTF indicated that the CNTF versions functionalized by 0.1 M and 0.2 M sulfanilic acid had the highest degree of functionalization (Figure 2b).

To confirm the effect of the functionalization, we examined the electric double-layer capacitance of the CNTF. Because CNTF has numerous nanopores and a moderately specific surface area, it operated as an electric double-layer capacitor (EDLC) without additional active material. When the voltage was applied to the CNTF immersed in an electrolyte, the electric charges accumulated at the interface between the CNTF and the electrolyte. Since the amount of electric charge was proportional to the interface area, the capacitance indicated an electrochemically active surface area of CNTF in a solvent [25]. Figure 2c shows the capacitive current obtained from CV curves versus scan rate in a three-electrode configuration with CNTF as a working electrode. (See the experimental section in detail.) The half slope of the graph of raw CNTF was the smallest among the CNTF types, meaning that raw CNTF showed poor water infiltration behavior and formed a small interface area between CNTF and water compared to the functionalized CNTF. It should be noted that a small amount of the attached sulfanilic acid group seemed to be effective to enhance water infiltration because the capacitance values of the functionalized CNTF were similar regardless of the degree of functionalization (Figure 2b). This result implied that SnO_2_ nanoparticles could not be grown in the inner nanopores of raw CNTF, wasting the surface area of the raw CNTF in an aqueous system.

Surface functionalization of CNTF with sulfanilic acid not only promoted the infiltration of water molecules but also increased the electrical conductivity of the CNTF. The doping effect of the sulfanilic acid group was observed in conducting polymers including polythiophene, polyaniline, and poly(3,4-ethylenedioxythiophene):poly(styrenesulfonate) (PEDOT:PSS) [26], which is the most widely used, commercial, conducting polymer. Likewise, it was postulated that the sulfonic acid group doped the CNTs and increased the electrical conductivity of CNTF. While chemical doping is a typical method of improving the electrical conductivities of CNTF [27], doping of CNTF using a sulfonic acid group was reported for the first time. In particular, it seemed to be a meaningful result to be doped from a functional group covalently bonded on CNT through a functionalization method such as diazotization. In contrast to the capacitance data, the enhancement of electrical conductivity after functionalization correlated with the degree of functionalization (Figure 2d). The 0.2S-CNTF had the highest electrical conductivity among the CNTF types studied in this work, which was consistent with the trend of the degree of functionalization. As the electrical conductivity increased by approximately 70% compared to that of the raw CNTF, the improved performance of electrochemical devices was expected. We also examined the mechanical properties of the CNTF before and after functionalization. The specific tensile strength of the functionalized CNTF decreased as the degree of functionalization increased (Figure 2d). Nevertheless, the strength was high enough to replace conventional current collector materials.

After growing the SnO_2_ nanoparticles in the aqueous solution, we obtained the ratio of SnO_2_ nanoparticles of SnO_2_@CNTF by comparison of linear density of CNTF before and after growing. Figure 2e shows the weight ratio of SnO_2_ nanoparticles of the samples. The weight ratio of CNTF and SnO_2_ nanoparticles of 0SC (SnO_2_@raw CNTF) was nearly 1:1. In contrast, that of CNTF and SnO_2_ nanoparticles of SnO_2_@functionalized CNTF was nearly 1:3, meaning that three times the number of SnO_2_ nanoparticles were grown on the surface of the functionalized CNTF compared to the raw CNTF. After growing the SnO_2_ nanoparticles on CNTF, the specific strength of the CNTF decreased as the linear density of the fiber increased (Figure 2f). Changes in the water infiltration behavior confirmed by EDLC measurements indeed affected the growth of the nanomaterials in an aqueous medium. The ratios of CNTF and SnO_2_ nanoparticles were almost the same, regardless of the degree of functionalization, which was similar to the result of the capacitance values. The 0.1CS and 0.2CS had a slightly larger amount of SnO_2_ than the others, implying that these two types would show the best capacity as an anode of LIB. A larger quantity of SnO_2_ was beneficial to enhance the energy storage performance of LIB because charge/discharge processes mainly occurred in SnO_2_. Considering that the proportion of the active material in the anode of a typical LIB is about 70 wt% [28], it is meaningful that 75–80 wt% of a whole anode consisted of the active material. Moreover, further optimization and studies can increase the fraction of the active materials.

**Figure 3:**
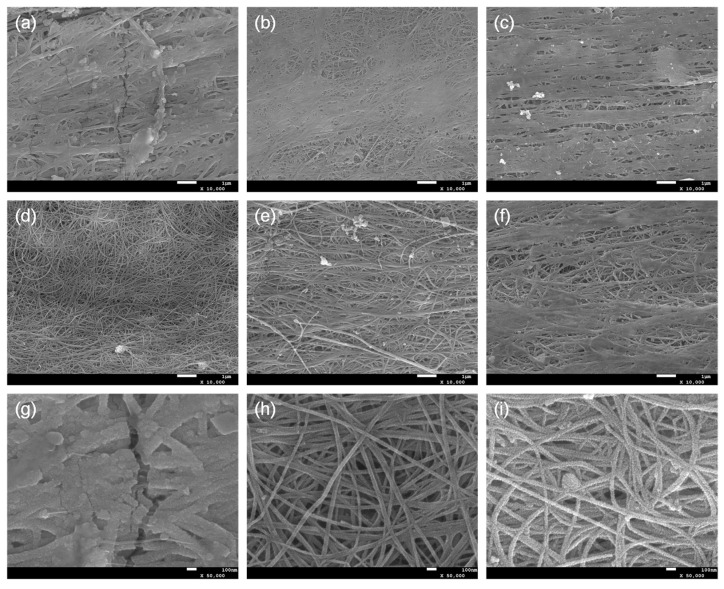
SEM images of (**a**) 0SC, (**b**) 0.025SC, (**c**) 0.05SC, (**d**) 0.1SC, (**e**) 0.2SC, and (**f**) 0.3SC. Magnified SEM images of (**g**) 0SC, (**h**) 0.1SC, and (**i**) 0.2SC.

SEM images showed the morphological characteristics of SnO_2_@CNTF (Figure 3). A different morphology of SnO_2_ nanoparticles was observed in each sample. Depending on the growth pattern of SnO_2_ nanoparticles, the SnO_2_@CNTF can be divided into (1) 0SC, (2) 0.025SC, 0.05SC, and 0.3SC, and (3) 0.1SC and 0.2SC. Since the morphology of CNTF was not changed by functionalization, the morphological differences of SnO_2_@CNTF are solely induced by the growth of SnO_2_ nanoparticles. The tendency well corresponds to the degree of functionalization. The 0SC had the lowest degree of functionalization and electrochemically active surface area. SnO_2_ nanoparticles formed a layer on the outer surface of the 0SC and clogged the pores between the CNT bundles (Figure 3a). The poor water infiltration behavior into the inner pores of the raw CNTF caused the selective growth of the SnO_2_ nanoparticles on the outer surface of CNTF. Additionally, the thickness of the SnO_2_ layer of 0SC was larger than those of other samples, inducing cracks on the layer. The 0.025SC, 0.05SC, and 0.3SC had a similar degree of functionalization (Figure 2d) and morphology (Figure 3b,c,f). The thin layer of SnO_2_ nanoparticles was formed, but pores between CNT bundles remained without blocking. The 0.1SC and 0.2SC had the highest degree of functionalization and profoundly ideal morphology (Figure 3d,e) because the SnO_2_ nanoparticles formed a thin coating on each CNT bundle. The evenly coated SnO_2_ formed a core-shell structure consisting of CNT bundles and a SnO_2_ layer. Compared to the SnO_2_@functionalized CNTF, the growth state of the SnO_2_@raw CNTF was unsuitable to be used in LIB anode for two reasons. (1) The amount of SnO_2_ of SnO_2_@raw CNTF was inevitably small because the nanoparticles could not be synthesized in the inner pores of CNTF. Since the main active material in this system was SnO_2_, a higher amount of SnO_2_ was more favorable to prepare high-performance LIB. Indeed, the ratio of SnO_2_ of 0CS was smaller than that of SnO_2_@functionalized CNTF despite the large thickness of the SnO_2_ layer of 0SC. That was because of the growth state of the SnO_2_ nanoparticles. (2) The aggregation of SnO_2_ nanoparticles was critical for LIB in terms of cyclability and energy density due to their large volume change during charge/discharge [29,30]. The volume change during lithiation of Sn alloy was more than 300% [31,32]. Active materials having a large volume change during the charging and discharging such as silicon or SnO_2_ need to be small to alleviate pulverization of the active materials [33,34]. Therefore, for preventing the formation of cracks and degradation of the performance of the anode, a uniform and thin coating was favorable.

**Figure 4:**
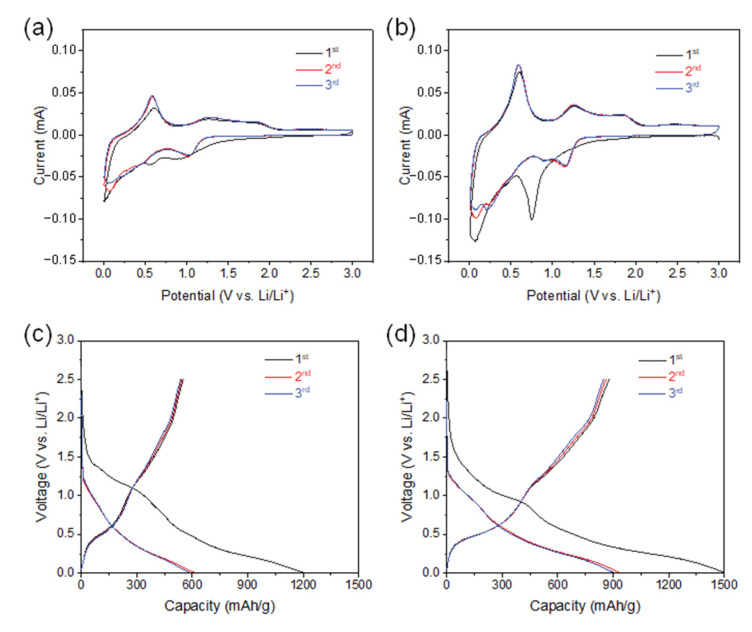
(**a**,**b**) CV curves in the potential ranging from 0.01 V to 3.0 V at a scan rate of 0.1 mV/s and (**c**,**d**) galvanostatic charge–discharge profiles at 100 mA/g of (**a**,**c**) 0SC and (**b**,**d**) 0.1SC.

We examined the anode performance of SnO_2_@CNTF by preparing 2032-type coin cells without additional conductive agents, binders, and current collectors. Stable solid–electrolyte interfaces (SEIs) are also critical in LIB anodes. Figure 4a,b shows CV curves of the SnO_2_@CNTF electrodes obtained over 0.01–3.0 V at a 0.1-mV/s scan rate. The first reduction peak at approximately 0.7 V versus Li/Li^+^ corresponded to SEI layer formation, and Li^+^ was readily accessible to SnO_2_ through this layer [35,36]. The reduction peak disappeared after the first cycle, indicating that the formation of the SEI layer was completed in the first cycle. The peak near 0 V versus Li/Li^+^ during reduction scan and near 0.6 V versus Li/Li^+^ during reverse anodic scan meant the lithiation and delithiation of the anode, respectively. The alloying and dealloying reactions were identical regardless of the SnO_2_@raw CNTF and SnO_2_@functionalized CNTF. The galvanostatic discharge curves also showed a gentle slope from 0.5 V–1.0 V versus Li/Li^+^, which disappeared after the first cycle (see Figure 4c,d). The charging capacity of the SnO_2_@CNTF was higher than the discharging capacity during the first cycle due to the irreversible formation of an SEI layer.

**Figure 5:**
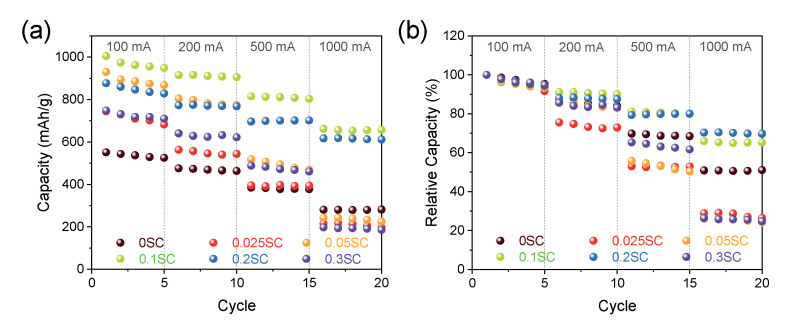
(**a**) Rate performance at a current density ranging from 100 mA/g to 1000 mA/g of SnO_2_@CNTF and (**b**) relative values of the capacity.

We measured lithium storage capacity for the SnO_2_@CNTF using galvanostatic charge/discharge at 100-, 200-, 500-, and 1000-mA/g rate conditions (see Figure 5). It should be noted that the discharge capacity described in this study was obtained by dividing the total weight of SnO_2_ and CNTF. That is, the discharge capacity in this work was underestimated compared to the discharge capacity calculated without the weight of a current collector, conductive carbon, and binder in other works because the mass ratio of active materials of the commercial LIB anodes was about 70%. In the low current density cycles, all SnO_2_@functionalized CNTF showed higher specific capacities than SnO_2_@raw CNTF due to the larger fraction of SnO_2_. Moreover, the uniform and thin coating on the functionalized CNTF provided larger reaction sites. We postulated that the differences in capacities of the SnO_2_@functionalized CNTF types were induced from the growth state of the SnO_2_ nanoparticles. The 0.1SC showed the highest specific capacity among the prepared samples. The capacity at the first cycle was 1005 mAh/g, which was 82% higher than 0SC (551 mAh/g). The conventional graphite anode usually has a capacity of 260 mAh/g when inactive materials including current collector, conductive carbon, and binder were included in the calculation of capacity. Therefore, the 10,095 mAh/g of capacity was remarkably high and improved the energy storage performance of full-cell-type batteries.

Interestingly, the specific capacities of the samples at a high current density were entirely different from the results at low current density. The 0.025SC, 0.05SC, and 0.3SC showed high specific capacities at a 100-mA/g rate, but the capacities significantly decreased as the current density increased. The specific capacities of the 0.025SC, 0.05SC, and 0.3SC at 1000 mA/g were 197 mAh/g, 225 mAh/g, and 186 mAh/g (in the fifth cycle), respectively; these were lower than that of 0SC (281 mAh/g) despite the higher ratio of SnO_2_ of SnO_2_@functionalized CNTF. In contrast, 0.1SC and 0.2SC exhibited high capacities at high current density (656 mAh/g and 612 mAh/g, respectively). The thin layer composed of SnO_2_ nanoparticles on the bundles of 0.1SC and 0.2SC was beneficial for volume expansion and electron transportation through the low conductive SnO_2_. On the other hand, the aggregation of the SnO_2_ nanoparticles on 0.025SC, 0.05SC, and 0.3SC not only induced the formation of cracks as the cycles progressed but also prohibited the electron transportation from the surface of SnO_2_ to the CNTF, leading to low performance and poor rate capability of the anode. The 0SC showed higher performance at high current density than 0.025SC, 0.05SC, and 0.3SC, despite the similar poor morphology of 0SC_,_ which was attributed to the high ratio of CNTF in the anode. Figure 5b shows relative values to the capacity at the first cycle, which was set to 1. The poor rate capability of the 0.025SC, 0.05SC, and 0.3SC can be verified more clearly at the relative values of rate performance. The capacity retention of the samples was about 30% compared to the first cycle. On the other hand, 0.1SC and 0.2SC exhibited higher capacity retention (~70%) than 0SC (~50%) despite the higher amount of SnO_2_ loading. As discussed in the morphological analysis, the SnO2@CNTF can also be divided into three groups here. When we considered the specific capacity at low current density and high current density simultaneously, the 0.1SC and 0.2SC were the best anode materials because of the high degree of functionalization, high conductivity, and ideal coating state. The rate capability and cycle stability of the SnO_2_ synthesized in this work were better than pure silicon particles [37].

**Figure 6:**
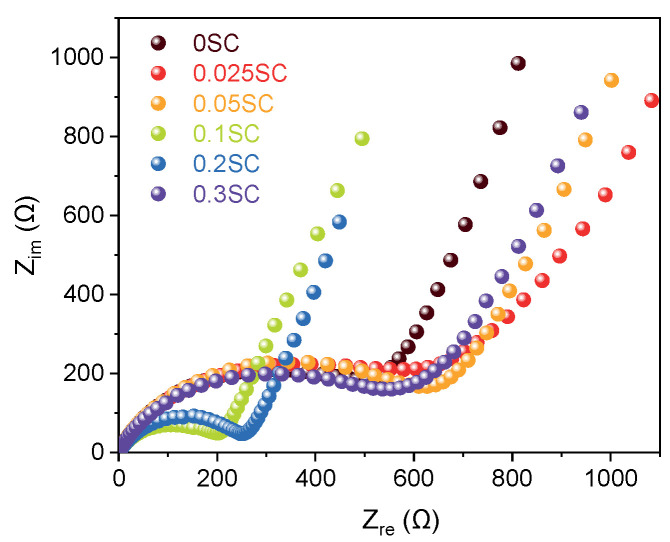
Nyquist plots of SnO_2_@CNTF obtained from EIS pattern.

Nyquist plots obtained from EIS of the SnO_2_@CNTF anodes provided information on the electrochemical behavior including solution resistance, charge transfer resistance, and Warburg impedance [38]. All samples exhibited similar solution resistance and Warburg impedance obtained by the slope at the low-frequency region (Figure 6) [15]. However, the diameters of the semicircles in the high-frequency region, corresponding to the charge transfer resistance at the interface between electrode and electrolyte, varied widely. Here, the results were also divided into three groups, as discussed above. The 0.1SC and 0.2SC showed obvious small resistance, indicating favorable charge transfer kinetics. It was well correlated with the high capacity and rate capability of the two anodes. We considered that the uniform and thin SnO_2_ coating on CNT bundles induced low charge transfer resistance of 0.1SC and 0.2SC. The charge transfer resistances of 0.025SC, 0.05SC, and 0.3SC were larger than 0SC as well as 0.01SC and 0.02SC. Considering that the 0.025SC, 0.05SC, and 0.3SC showed lower rate capability than 0SC, the Nyquist plots were in good agreement with the rate capability.

## 4. Conclusions

In this work, we prepared a conductive agent, binder, and current collector-free anode of LIB based on functionalized CNTF. Due to the hydrophobic nature of CNTF, sulfonic acid groups were introduced on the surface of CNTF to grow an active material in an aqueous system. The sulfonic acid groups not only improved the water infiltration into the nanopores of CNTF but also increased the electrical conductivity of the CNTF, which made the functionalized CNTF suitable to be used in LIB. The concentration of precursors during functionalization significantly influenced the degree of functionalization and SnO_2_ nanoparticle growth. Functionalized CNTF treated by 0.1 M and 0.2 M of sulfanilic acid showed the best characteristics including electrical conductivity, degree of functionalization, and growth morphology of SnO_2_ nanoparticles. Ideal morphology consisting of CNT bundles and a thin layer of the SnO_2_ nanoparticles was obtained for the functionalized CNTF because the SnO_2_ nanoparticles distributed very uniformly without clogging the nanopores between the CNT bundles. The CNTF treated by 0.1 M and 0.2 M of sulfanilic acid exhibited the highest specific capacity and rate capability, which was supported by the low charge transfer resistance observed in the Nyquist plot. Overall, the degree of functionalization, morphology of nanoparticle growth, rate capability of the anode, and charge transfer resistance were closely related to each other. Therefore, optimized surface functionalization is the key issue in the preparation of LIB electrodes based on CNTF. We only addressed the issues on the functionalization in this study, but further works on reducing the diameter of CNT bundles can increase the ratio of SnO_2_, leading to higher performance of SnO_2_@CNTF based LIB anode.

## Data Availability

The data presented in this study are available on request from the corresponding author.
